# A Case of Recurrent Thrombotic Microangiopathy Caused by Hypertensive Urgency

**DOI:** 10.7759/cureus.3235

**Published:** 2018-08-30

**Authors:** Farman Ali, Aman Ullah, Waseem Amjad, Tanureet Kochar, Frank H Annie, Ali Farooq

**Affiliations:** 1 Medicine, St. John Hospital and Medical Center, Detroit, USA; 2 Internal Medicine, St. Joseph Mercy Oakland Hospital, Pontiac, USA; 3 Digestive Diseases, Mercy Medical Center, Baltimore, USA; 4 Internal Medicine, West Virginia University/Charleston Area Medical Center, Charleston, USA; 5 Cardiology, West Virginia University/Charleston Area Medical Center, Charleston, USA

**Keywords:** thrombotic microangiopathy, hypertensive urgency, thrombotic thrombocytopenic purpura

## Abstract

A 26-year-old man presented to the emergency room with abdominal pain, nausea, and vomiting for four days. His medical history was significant for hypertension and end-stage renal disease managed with hemodialysis. He had been noncompliant with the antihypertensive regimen which included nifedipine, hydralazine, and spironolactone. At presentation, his blood pressure was 231/123 mmHg. Laboratory workup showed white blood count 17.3 × 109/L (normal range: 4.5 to 11.0 × 109/L), hemoglobin 7.8 gm/dL (normal range: 13.5 to 17.5 g/dL), platelet count 46 × 109/L (normal range: 150 to 400 × 109/L), reticulocyte count 7.8%, total bilirubin 1 mg/dL (normal range: 0.1 to 1.2 mg/dL), lactate dehydrogenase 1,235 U/L (normal range: 140 to 280 U/L), haptoglobin < 10 mg/dL, and a direct Coomb's test was negative. Numerous schistocytes were identified on the peripheral blood smear. The patient was diagnosed with thrombotic microangiopathy secondary to severe hypertension and was started on intravenous nicardipine. With appropriate blood pressure control, hematological parameters improved with normalization of the platelet count within 10 days. Notably, the patient had one similar episode of hypertension-induced thrombotic microangiopathy within a period of the last three months and ADAMTS-13 (a disintegrin and metalloprotease with thrombospondin type 1 motif 13) activity was normal on his previous admission.

## Introduction

Thrombotic microangiopathy (TMA) is a clinical syndrome that is characterized by microangiopathic hemolytic anemia, thrombocytopenia, and end-organ damage [1].

Several causes of TMA have been described, including thrombotic thrombocytopenic purpura (TTP), hemolytic uremic syndrome, malignancy, drugs, and severe hypertension. The threshold of blood pressure beyond which TMA occurs remains unknown; however, TMA appears to occur more frequently when systolic and/or diastolic blood pressures exceed 200 mmHg and 100 mm Hg, respectively [2]. The initial clinical presentation of hypertension-induced TMA is indistinguishable from TTP-induced TMA, except for the presence of severe hypertension. However, a history of hypertension, significant renal impairment, relatively modest thrombocytopenia, and lack of a severe ADAMTS-13 (a disintegrin and metalloprotease with thrombospondin type 1 motif 13) deficiency (activity > 10% in hypertension-induced TMA) serve as useful clues for diagnosis of hypertension-induced TMA [3]. We report a case of recurrent TMA caused by a hypertensive urgency in a patient with end-stage renal disease (ESRD).

## Case presentation

**Figure 1:**
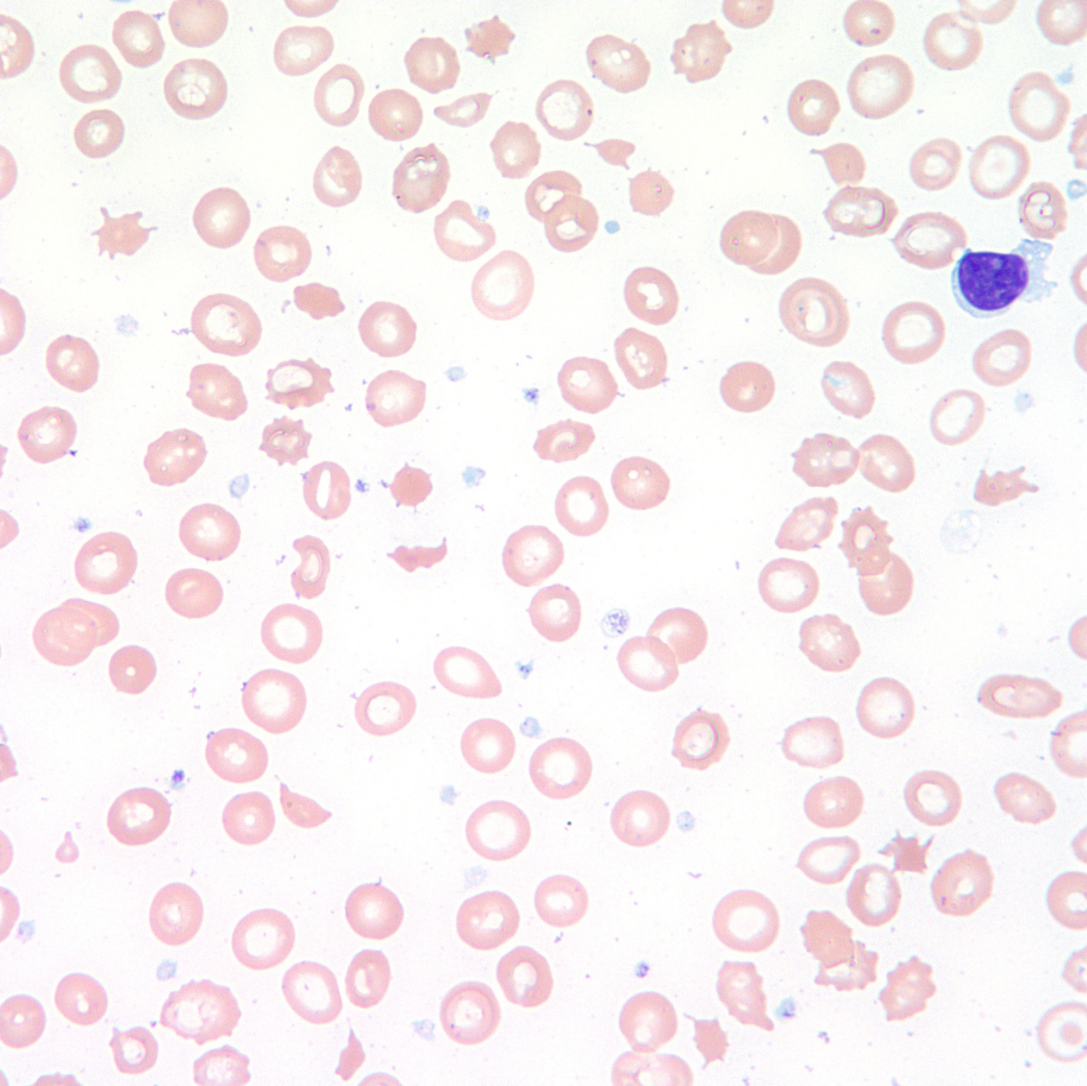
Peripheral blood smear showing schistocytes

A 26-year-old African American male with a past medical history of hypertension, end-stage renal disease managed by hemodialysis presented to the emergency department with complaints of abdominal pain, nausea, and vomiting. He had been noncompliant with his antihypertensive medications which included nifedipine, hydralazine, and spironolactone. On presentation, the patient’s blood pressure was 231/123 mmHg. Laboratory workup showed a white blood count of 17.3 × 109/L (normal range: 4.5 to 11.0 × 109/L), hemoglobin 7.8 gm/dL (normal range: 13.5 to 17.5 g/dL), platelet count 46 × 109/L (normal range: 150 - 400 × 109/L), reticulocyte count 7.8%, total bilirubin 1 mg/dL (normal range: 0.1 to 1.2 mg/dL), lactate dehydrogenase 1,235 U/L (normal range: 140 to 280 U/L), haptoglobin < 10 mg/dL, and direct Coomb's test was negative. Numerous schistocytes were identified on a peripheral blood smear (Figure 1). 

## Discussion

Hypertensive urgency can affect up to 1% of all hypertensive patients and accounts for 3% of all emergency room visits and 25% of all medical emergencies [4-5]. Although malignant hypertension is a common reason for patients to visit the emergency department, it rarely causes TMA. In malignant hypertension, the autoregulatory mechanisms controlling blood pressure fails. This results in damage to the vascular endothelium and obliteration of the vascular lumen which leads to fibrin clots and platelets clots within the microvascular network and subsequently causes hemolysis of red blood cells and platelet consumption in this process [6].

The initial clinical presentation of hypertension-induced TMA is indistinguishable from TTP-induced TMA, except for the presence of severe hypertension. TTP is characterized by the presence of unexplained microangiopathic hemolytic anemia, thrombocytopenia, fever, acute renal impairment, and neurological deficits, although this pentad is rare (present in < 5% cases).

TTP is a disorder of von Willebrand factor (VWF) proteolysis, which is caused by either a congenital deficiency or an autoimmune-acquired, antibody-mediated destruction of plasma metalloprotease, ADAMTS-13. VWF and platelets have the tendency to form aggregates in high shear stress in the microcirculation that is mitigated by ADAMTS-13. In absence of ADAMTS 13 activity, VWF-platelets aggregate and form microvascular thrombosis of TTP. Documenting severe ADAMTS-13 deficiency (activity < 10%) supports the diagnosis of TTP [7]. However, TTP is a clinical diagnosis and measurement of ADAMTS-13 activity is not required to initiate initial management with plasmapheresis as it is a medical emergency with a very high mortality rate if left untreated. Therefore, it is important to diagnose TTP promptly in appropriate clinical settings and treat accordingly. However, a history of hypertension, significant renal impairment, relatively modest thrombocytopenia, and a lack of severe ADAMTS-13 deficiency (activity > 10% in hypertension-induced TMA) serve as useful clues for the diagnosis of hypertension-induced TMA. Also, fever is uncommon in patients with TMA caused by malignant hypertension. Common presenting symptoms in these patients can be headache, blurry vision, dizziness, nausea, vomiting, and abdominal pain, as was the case in our patient [3].

Inability to reliably differentiate TTP from malignant hypertension-induced TMA clinically presents as a dilemma for clinicians in deciding whether or not to initiate plasma exchange.

## Conclusions

TMA is a clinical syndrome that is characterized by microangiopathic hemolytic anemia, thrombocytopenia, and organ injury. Severe hypertension can induce thrombotic microangiopathy, mimicking that induced by TTP. Plasma exchange is not utilized for management of hypertension-induced thrombotic microangiopathy; instead, blood pressure control is the treatment of choice. The absence of fever, modest thrombocytopenia, and elevated blood pressure favors the diagnosis of hypertension-induced TMA in comparison with TTP.
